# Gut microbiota disturbances and protein-energy wasting in chronic kidney disease: a narrative review

**DOI:** 10.1007/s40620-022-01560-1

**Published:** 2023-01-23

**Authors:** Fabiola Martín-del-Campo, Carla Maria Avesani, Peter Stenvinkel, Bengt Lindholm, Alfonso M. Cueto-Manzano, Laura Cortés-Sanabria

**Affiliations:** 1 Unidad de Investigación Médica en Enfermedades Renales, Hospital de Especialidades, Centro Médico Nacional de Occidente, Instituto Mexicano del Seguro Social, Guadalajara, Jalisco Mexico; 2 Division of Renal Medicine and Baxter Novum, Department of Clinical Science, Technology and Intervention, Karolinska Institutet, M99 Karolinska University Hospital Huddinge, 14186 Stockholm, Sweden

**Keywords:** Gut microbiota, Protein-energy wasting, Chronic kidney disease, Dialysis, Leaky gut

## Abstract

Protein-energy wasting (PEW) is common in patients with chronic kidney disease (CKD) and is associated with increased morbidity and mortality, and lower quality of life. It is a complex syndrome, in which inflammation and retention of uremic toxins are two main factors. Causes of inflammation and uremic toxin retention in CKD are multiple; however, gut dysbiosis plays an important role, serving as a link between those entities and PEW. Besides, there are several pathways by which microbiota may influence PEW, e.g., through effects on appetite mediated by microbiota-derived proteins and hormonal changes, or by impacting skeletal muscle via a gut-muscle axis. Hence, microbiota disturbances may influence PEW independently of its relationship with local and systemic inflammation. A better understanding of the complex interrelationships between microbiota and the host may help to explain how changes in the gut affect distant organs and systems of the body and could potentially lead to the development of new strategies targeting the microbiota to improve nutrition and clinical outcomes in CKD patients. In this review, we describe possible interactions of gut microbiota with nutrient metabolism, energy balance, hunger/satiety signals and muscle depletion, all of which are strongly related to PEW in CKD patients.

**Graphical abstract:**

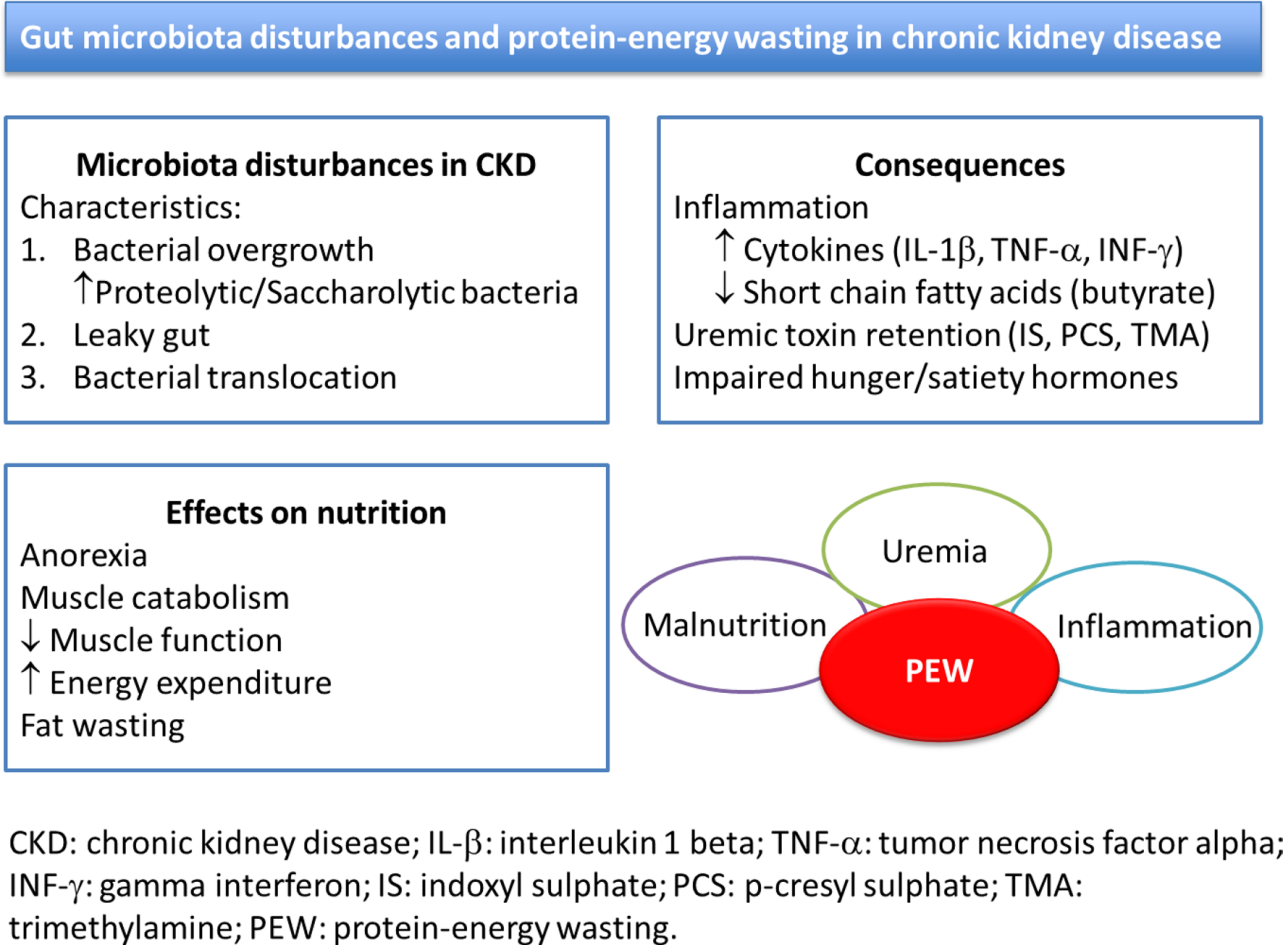

## Introduction

The gut has recently received increased attention as a potential target for preventive and therapeutic interventions aimed at decreasing the production, release and systemic absorption of gut-microbiota-derived uremic toxins in patients with advanced chronic kidney disease (CKD). The hypothesis is that these therapeutic interventions could potentially contribute to the prevention of metabolic and cardiovascular complications in this vulnerable population [1].

Microbiota disturbances have been linked to occurrence, progression and development of a range of complications in many chronic diseases, such as inflammatory bowel diseases [2], diabetes [3], neurological disorders [4] and cancer [5], and to acute infections not only in the gut but also in remote organs like the lungs during COVID-19 infection [6]. Trillions of commensal bacteria dynamically interact with the host through the intestinal epithelial cells, modulating local and systemic immunological functions; these interactions are related to nutrient digestion, absorption, metabolism and clearance of waste products, consequently affecting essentially all nutrition processes in the body [7]. Understanding the pathways and mechanisms by which nutritional balance could be modified and likely regulated by the gut microbiota is therefore important in identifying new potential therapeutic targets to prevent and treat complex nutritional disturbances.

It is now well recognized that protein-energy wasting (PEW) is a main complication in patients with advanced CKD that is associated with higher morbidity and mortality and lower quality of life [8, 8]. PEW is a complex syndrome characterized by loss of body muscle and fat stores, associated with inflammation and with the patient’s uremic conditions [10]. In this review, we describe possible interactions of gut microbiota with nutrient metabolism, energy balance, hunger/satiety signals and muscle depletion, all of which are closely related to PEW in CKD patients.

## The uremic gut

The gut is an organ with multiple functions including absorption of nutrients, while at the same time preventing access of inflammatory and antigenic compounds into the body through mechanical, immunological and ecological external defense barriers. In addition, the gut is the host of a complex microbiota that is a source of numerous essential as well as potentially harmful metabolites. The human gut contains most of the microbial biomass in the body, with trillions of commensal bacteria, from more than 1000 different operational taxonomic units, accounting for 10 times more cells and a 150 times larger gene pool than the host [11]. The composition of gut microbiota varies according to age, geography, health conditions, lifestyle, and genetics, among many other factors; however, it is relatively stable in healthy individuals, and exhibits certain characteristics associated with health especially when it is mainly dominated by Firmicutes, Bacteroidetes, Actinobacteria and Proteobacteria bacteria phyla [12]. On the other hand, when the fine tuned balance between these microbes is altered, leading to gut dysbiosis, it may contribute to the development and progression of several diseases affecting not only the gut but also distant organs and systems.

It is now well established that CKD patients exhibit alterations in gut microbiota, with an increase in bacterial total counts of predominantly proteolytic bacteria. Compared to healthy individuals, patients have a lower α-diversity (species richness), as well as a decreased relative abundance of bacteria of the phyla Firmicutes and Actinobacteria, while Proteobacteria is increased. *Enterococcus* and *Clostridium* families are enriched in CKD, whereas the *Prevotella, Coprococcus, Megamonas, Sutterella, Enterobacter, Acidaminococcus, Dorea*, and *Roseburia* families are more abundant in healthy individuals [13]. When bacterial families are classified according to their enzyme characteristics, CKD patients have greater abundance in urease, uricase, tryptophanase (indole-forming enzyme) and hydroxyphenylacetate decarboxylase (p-cresol-forming enzyme) families, as well as a decrease in those with phosphotransbutyrylase and butyrate kinase (butyrate-forming enzymes) compared to healthy controls [14]. However, the latter studies did not always consider factors such as sex, age, comorbidities, dietary intake, environmental conditions and genetic aspects that may also influence gut microbiota in the CKD patient; this is supported by studies showing that gut microbiota in dialysis patients did not differ from that of healthy household contacts with similar dietary characteristics [15, 15].

Gut microbiota of CKD patients may not only differ from healthy people, but also according to the type of renal replacement therapy. A metagenomic analysis of microbiota in pediatric peritoneal dialysis (PD) patients showed lower relative abundance of Firmicutes and Actinobacteria phyla, and greater abundance of Bacteroidetes phyla, Proteobacteria phyla, and *Enterobacteriaceae* family than in hemodialysis (HD) patients. Children receiving PD and renal transplantation were reported to have a lower relative abundance of *Bifidobacteriacea* as well as lower α-diversity compared to healthy controls, however, the mean concentrations of uremic toxins did not differ among modalities [17]. Differences in gut microbiota have also been observed between adult non-dialysis, PD and HD patients, although whether PD or HD patients have better or worse microbiota is controversial, and may be associated with differences in metabolic and clinical variables [18, 18]. The microbiota in pediatric patients seems to show fewer differences in metabolic and clinical variables compared to adult populations, perhaps due to the different etiology of CKD in children [17].

Possible factors associated with these differences include characteristics of the renal replacement therapy itself, such as the hemodynamic changes in HD, the intestinal absorption of glucose from the dialysate during the PD procedure, or the use of immunosuppressive drugs in renal transplantation. Further research is necessary to confirm differences between these treatment modalities, to identify mechanisms by which a specific renal replacement therapy may affect the gut microbiota, and to analyze the potential clinical consequences of such changes.

In uremia, increased concentrations of nitrogen waste products, decreased intake of fiber, presence of malnutrition/malabsorption and constipation, and medication use, are some of the most common factors that were reported to be associated with bacterial overgrowth and dysbiosis in the gut [20]. Of importance, CKD patients have a high pill burden due to multiple comorbidities [21], and chronic use of some of the commonly used medications in CKD may alter the gut microbiota: (a) *phosphate binders* affect the intestinal environment, increasing gastrointestinal symptoms, and may bind to harmful molecules—other than phosphate—such as p-cresol, endotoxin, advanced glycation end products, bile acids, and oxalates, but also to beneficial molecules as vitamin K, folic acid, and short chain fatty acids (SCFAs), hence modifying nutrient absorption and gut microbiota [22]; (b) *antibiotics* lead to a dramatic impairment of the gut microbiota with a decrease in bacterial diversity and loss of different taxa, and the inappropriate use or overuse of antibiotics increases the risk of antibiotic-resistant infections, epithelial barrier disruption, and development of metabolic and immune diseases [23]; (c) *oral iron* medication promotes dysbiosis, thereby decreasing SCFA-forming bacteria and increasing proteolytic bacteria, and moreover, a high iron load promotes replication and virulence of pathobionts such as *Salmonella*, *Shigella*, *Campylobacter* or *Citrobacter*. In addition, luminal iron load induces the generation of reactive oxygen species, causing oxidative stress and intestinal epithelial damage [24]. All the above-mentioned mechanisms favor increased uremic toxin production, translocation of bacterial compounds including lipopolysaccharide, (LPS), and decreased SCFA synthesis [20].

Bacterial overgrowth, along with an increase in sympathetic activity, accumulation of nitrogen waste compounds, and the presence of stress and intestinal inflammatory diseases, all favor the loss of intestinal wall integrity by damaging the apical binding complex and decreasing survival of epithelial cells [25]. Apical binding disruptions occurring by several mechanisms may increase intestinal permeability, leading to a “leaky gut” and thereby enabling bacterial translocation. When urea reaches the intestinal lumen, it is metabolized by urease-containing bacteria into ammonium that is subsequently hydrolyzed into caustic ammonium hydroxide, which erodes the epithelial wall. The latter stimulates influx of inflammatory leukocytes, triggering retraction and endocytosis of apical binding transcellular proteins (occludin and claudin) as a consequence of increased local production of cytokines [26]. On the other hand, bacterial tryptophanase decreases host tryptophan bioavailability, and promotes an enteric rise of the levels of serotonin, a tryptophan metabolite, that may further worsen gut barrier dysfunction [27].

**Fig. 1:**
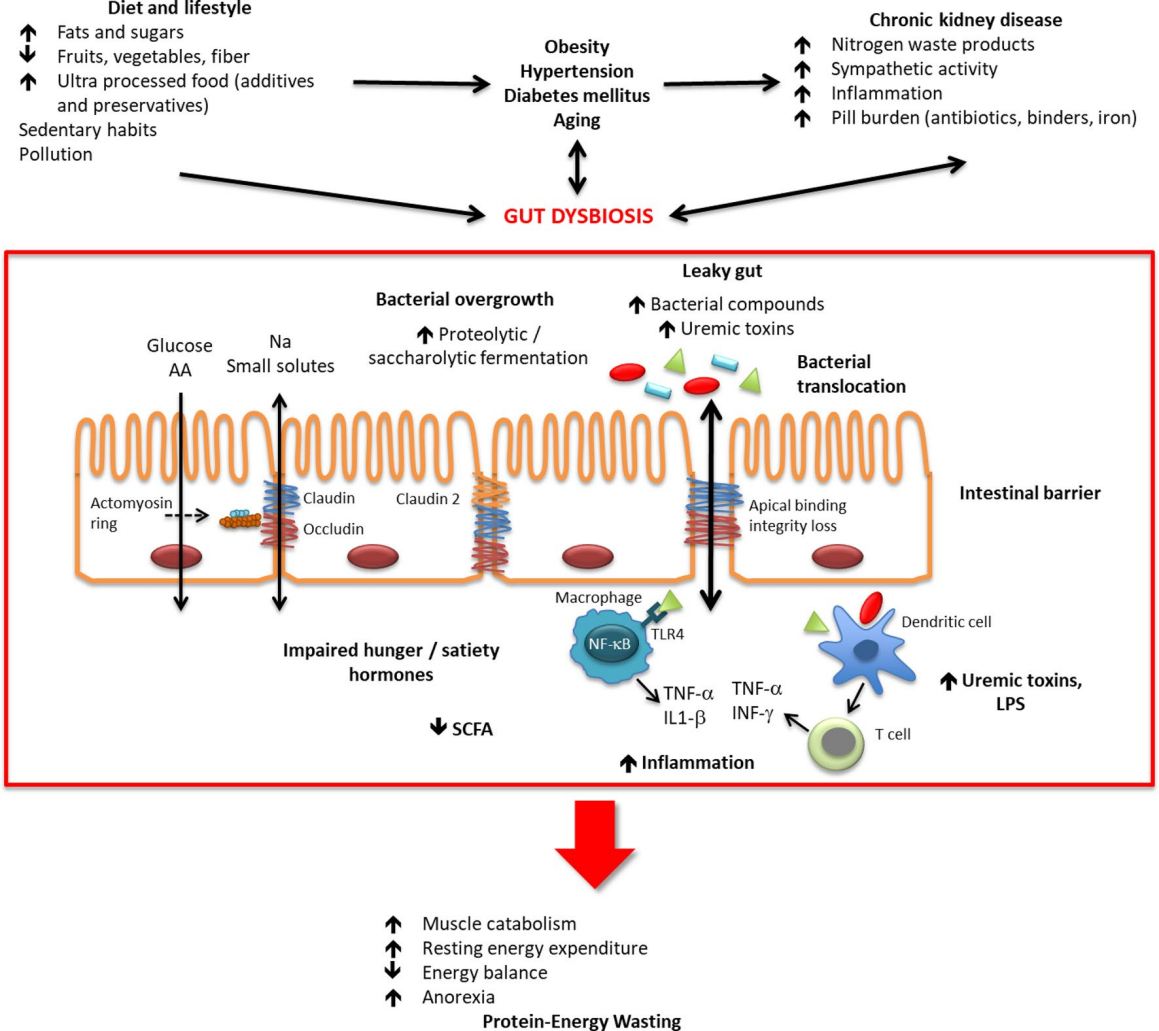
Complex interactions between factors promoting gut dysbiosis and occurrence of protein-energy wasting in CKD patients

Bacterial translocation is recognized as a cause of local and systemic inflammation [28]. Once in the circulation, bacterial endotoxins join the LPS protein binding sites, forming a complex protein, which interacts with myeloid differentiation factor-2in the toll-like receptor 4 anchored to the CD14, thereby stimulating the transcription of nuclear factor kappa B (NF-κB), which in turn leads to increasing production and release of inflammatory cytokines as tumor necrosis factor-α (TNFα) and IL-1β [29] [25]. Endotoxin also mediates endothelial damage by boosting monocyte recruitment, transforming macrophages into foam cells and activating coagulant activity [1]. Additionally, pathogenic bacteria stimulate dendritic cells, activating T-cell response and increasing the production of TNFα and interferon-γ (INF-γ) [29]even more. A summary of findings related to the uremic gut is presented in Fig. 1.

## Gut microbiota in food deprivation diseases

As far as we know, there are few studies focusing on links between PEW in CKD and microbiota characteristics. However, recent studies in other malnutrition syndromes suggest that microbiota could play a role in its development, and modification of the microbiota could be an opportunity to positively change the course of processes leading to PEW and thereby improve clinical outcomes.

Children with Kwashiorkor present low bacterial diversity in the gut, as well as an increase in Proteobacteria phyla and *Streptococcus*, and a decrease in *Prevotella* and *Bacillus* genus [30]. Malnourished Iranian children showed similar changes, with an increase in Proteobacteria phyla (*Escherichia*, *Streptococcus*, *Veillonella*, *Shigella*), and a decrease in *Roseburia*, *Faecalibacterium* and *Butyrivibrio* genus, along with increased severity of malnutrition [31]. Moreover, analysis of genome-scale metabolic models of gut microbiota shows that malnourished children have reduced levels of essential amino acids and lower metabolic capability to produce several non-essential amino acids, compared to healthy children [32].

Patients with anorexia nervosa present lower microbiota diversity, lower counts of anaerobic butyrate-forming bacteria, and lower SCFA concentrations, as well as increased counts of proteolytic and mucin-degrading bacteria, compared to healthy subjects. Weight gain in anorectic patients is associated with favorable changes in gut microbiota, with an increase in α-diversity, Firmicutes and Actinobacteria phyla, and *Bifidobacteria* and *Ruminococcus *spp [33]. It is possible that modifications in gut microbiota in food deprivation states could be the result of an adaptive response to optimize food transformation in low calorie diets. However, it should be noted that the etiology of PEW in CKD has significant differences compared to the aforementioned conditions, which are affected by factors such as extremely low dietary intake, poor quality of diet and poor hygiene.

Although CKD patients are exposed to many conditions other than low food intake that could lead to malnutrition (endocrine disorders, comorbidities, dialysis-related factors, metabolic acidosis, inflammation, and oxidative stress), it is possible that dysbiosis caused by inadequate dietary intake with lack of fibers may play a major role for the development of PEW. However, studies in this regard are scarce and implications of gut microbiota alterations on PEW development should be interpreted cautiously, particularly given the lack of longitudinal analyses. In a case–control study in HD patients, similar findings were seen, as patients with PEW presented lower α-diversity, *Prevotella* and *Faecalibacterium* genus abundance, as well as an increase in *Enterococcus*, *Scardovia*, *Bifidobacterium*, *Anaerovorax*, *Collimonas* y *Akkermansia* genus abundance compared to patients with normal nutrition by subjective global assessment [34]. In dialysis patients, muscle indicators such as mid-arm muscle area, and handgrip strength were positively correlated with *Roseburia*, *Phascolarctobacterium* and *Coprococcus* genus and negatively with Escherichia genus [35].

## Nutrient metabolism in the gut: changes induced by uremia

The main determinant of bacterial metabolism is nutrient availability, particularly the disposable carbohydrate/protein ratio in the colon, which regulates the proteolytic and saccharolytic fermentation in the gut. Fermentable carbohydrates are the leading energy source, whereas proteins and amino acids are used essentially to increase biomass, however, protein fermentation (putrefaction) increases when carbohydrate availability is low [7, 36]. In CKD patients, the high concentrations of urea and nitrogen waste products reaching the gut through the intestinal fluids, together with greater bioavailability of amino acids and peptides related to malabsorption and/or intestinal edema, stimulate overgrowth of proteolytic bacteria, which in turn, increases production of uremic toxins. Additionally, low intake of fermentable carbohydrates is common in CKD patients, as foods rich in potassium and phosphorus such as fruits, vegetables and legumes that have a high fiber content are frequently restricted, thus decreasing the fermentation by saccharolytic bacteria and consequently the production of SCFAs [20, 36].

### Bacterial uremic toxins and their role in PEW: impact of the gut-muscle axis in CKD

There is growing evidence of a special communication network between gut microbiota and skeletal muscle (size, composition and functionality), particularly in aging and cancer cachexia settings, the so-called *gut-muscle axis* [37]. In CKD patients, intestinal bacterial metabolism generates uremic toxins, such as p-cresyl sulphate (PCS), indoxyl sulphate (IS), phenylacetic acid, indole-3-acetic acid (IAA), hippuric acid (HA), and trimethylamine that promote local and systemic pro-inflammatory activity and associate with progression of cardiovascular disease and CKD [1]. Gut-derived uremic toxins and bacterial endotoxins may enhance PEW development, regardless of their well-known inflammatory effect, particularly because of their effect on skeletal muscle. Nonetheless, this effect of gut dysbiosis has been poorly explored (Table 1).

**Table 1:** Gut derived compounds related to dysbiosis and their possible effect on PEW development in CKD

Compounds	Association with CKD	Effect on PEW	References
Inflammatory cytokines	↑	Anorexia Muscle and fat wasting Increased resting energy expenditure	[7, 61]
p-Cresyl sulphate	↑	Lipolytic Muscle fat infiltration	[32]
Indoxyl sulphate	↑	Muscle catabolism and atrophy	[34, 35]
Glycine-conjugated compounds	↑	Decreased muscle function and physical performance	[36–38]
Acrolein	↑	Muscle wasting	[44]
Endotoxins	↑	Muscle wasting and atrophy Increased resting energy expenditure Anorexia	[45, 46]
SCFAs	↓	Inflammation Energy imbalance Protein catabolism	[6, 48, 49] [50]
Appetite regulation peptides	↑↓	Anorexia	[51, 53, 54, 56]

*SCFAs* short chain fatty acids

In rats, administration of PCS originating from bacterial degradation of tyrosine and phenylalanine promoted insulin resistance, and loss and redistribution of fat; of note, body composition changes included a decrease in white fat, while there was an increase in liver ectopic lipid content and muscle fat infiltration [38], which is also associated with mobility impairment [39]. In the same study, PCS decreased lipogenesis and increased lipolysis in human adipocytes, which suggests a catabolic effect affecting fat mass [38].

IS is an aromatic compound that derives from bacterial tryptophan metabolism. In CKD murine models, IS accumulates in skeletal muscle and promotes muscle loss and atrophy by inhibiting protein synthesis, cell proliferation and viability, increasing oxidative stress and inflammation, impairing mitochondrial function, and accelerating amino acid degradation [40]. In addition, as reported in the same paper, serum concentrations of IS in PD patients were found to be associated with decreased muscle mass, showing further subsequent reductions following 2 years of dialysis [40]. In an in vitro study, myoblast cells were treated with different gut-derived uremic toxins: IS, and to a lesser extent IAA, significantly inhibited cell proliferation, but only IS increased oxidative stress in a dose-dependent manner, and IS also increased inflammation, as well as myostatin and atrogin-1, which are negative regulators of muscle growth involved in muscular atrophy [41].

Gut bacterial metabolites have also been implicated as factors affecting muscle function and physical performance in both young and older healthy adults; hydrocinnamate, cinnamoylglycine, indolepropionate and HA were negatively associated with lower extremity muscle quality, an indicator of muscle function [42], and physical performance [43]. HA is a toxic solute derived from bacterial degradation of aromatic compounds such as aromatic amino acids, preservatives with benzoic acid, and contaminants as toluene or polyphenols, which inhibit glucose utilization in muscle cells, thus potentially increasing muscular weakness in CKD patients [44]. It has controversial effects, since high levels of HA derived from dietary antioxidant polyphenols, as catechin (green and black tea) or chlorogenic acid (coffee) have positive effects on muscle metabolism and performance [45] by promoting myogenic differentiation and myofiber regeneration [46]. It is possible that diverse dietary sources, gut dysbiosis and interactions with the uremic host environment could lead to the observed differences [47].

Polyamines, for their part, are metabolites from protein fermentation in the gut, which are required for normal cell proliferation and differentiation; in muscle cells, regulation of polyamines is associated with hypertrophy and restoring muscle after injury, and a shift in polyamine equilibrium can lead to dysregulation in muscle metabolism and growth [48]. In CKD patients, putrescine levels are higher, while spermidine and spermine levels are lower compared to subjects with normal kidney function. Additionally, there was an increase in spermidine and spermine degradation into acrolein, a toxic compound [49], which has inhibitory effects on myogenic differentiation and induces muscle wasting in animal models [50].

Finally, endotoxin or LPS, a phospholipid of the bacterial outer membrane, increases systemic inflammation, which induces muscle and fat catabolism and metabolic derangements (insulin resistance). Peripheral and central nervous system inflammatory effects also mediate LPS-induced muscle catabolism. Skeletal muscle has receptors for pathogen-associated molecules, catabolic hormones and proinflammatory cytokines, so that LPS may act by different action mechanisms to promote muscle wasting and atrophy; the most recognized mechanisms involve activation of the ubiquitin–proteasome and autophagy-lysosome pathways and down-regulation of insulin-like growth factor-1 [51]. Moreover, LPS exerts inflammatory effects in the central nervous system by stimulation of the melanocortin system that increases resting energy expenditure and weight loss, as well as by activation of hypothalamus NF-κB that increases anorexia and muscle wasting [52].

Recently, it has been shown that intestinal infections may activate the NLRP3 inflammasome stimulating the innate and adaptive immune responses, which in turn may increase muscle atrophy and wasting via caspase-1 activation [53]. Moreover, inflammasome signaling may be associated with hyperfiltration in some kidney diseases [54].

### SCFA saves energy in the gut and has anti-inflammatory effects

Dietary fibers do not per se provide energy directly to humans, as we are unable to digest and absorb them, however, colonic bacteria can metabolize resistant starch and fermentable fibers to butyrate, propionate and acetate SCFAs, as well as to lactate and various gases. Butyrate is the main energy source of epithelial cells and promotes integrity of the intestinal wall; propionate is mostly metabolized in the liver, as a precursor in gluconeogenesis and lipogenesis; a proportion of the acetate is metabolized to glutamine (main energy source of the small intestine), while muscles can metabolize another part to obtain energy. SCFAs and other compounds derived from the metabolism of fibers may provide substantial amounts of energy, 1.5–2 kcal/g of fiber, contributing to preserve the “lost” energy from the small intestine, thus enhancing energy homeostasis. It has been proposed that it could be a healthy opportunity to conserve energy from the diet in poor communities or in individuals with compromised diets due to restrictions such as those recommended in CKD patients [7]. It has also been observed that obese subjects have higher relative abundance of Firmicutes and a lower amount of Bacteroidetes compared to lean subjects, and an increase in Bacteroidetes was associated with greater weight loss during the diet period [55]. In adolescents with obesity, the Firmicutes/Bacteroidetes ratio, the relative abundance of Bacteroidetes and Actinobacteria phylum as well as seven different genus bacteria (*Actinomyces*, *Odoribacter*, *Oscilospira*, *Bifidobacterium*, *Streptococcus*, *Bacteroides*, *Faecalibacterium*) were positively associated to total, visceral, subcutaneous and hepatic fat content, and to SCFA concentrations, suggesting that their gut microbiota profile has a better ability to extract energy from carbohydrates [56]. Although SCFAs are associated with efficient energy harvest in the gut and weight gain, it seems, on the basis of rodent models, that these effects can be linked to changes in energy utilization and expenditure, as SCFAs inhibit fat accumulation, enhance adaptive thermogenesis and fat oxidation (increase energy expenditure), and decrease markers of protein catabolism [57]; these effects could be associated with improvements in body composition.

On the other hand, SCFA modulates the inflammatory response, suppressing synthesis and release of cytokines in neutrophils, macrophages and other cells, and suppresses adhesion molecule expression in endothelial cells. Butyrate has the greatest anti-inflammatory effect, as it suppresses LPS-stimulated production of TNFα, IL-6 and nitric oxide, and increases IL-10 release. The main anti-inflammatory mechanism of action is the attenuation of histone deacetylase enzyme activity, which stimulates the increase in histone and nonhistone protein acetylation, such as that of NF-κb, modulating gene expression of cytokines [58].

### Gut microbiota is related to appetite and nutrient intake control

The causes of anorexia, which further worsens PEW in CKD patients, include a range of factors such as retention of uremic solutes due to loss of residual renal function, inflammation, effects of the dialysis procedure, dietary restrictions, taste abnormalities, depression, gastrointestinal symptoms, comorbidities, pill burden, as well as alterations in hunger-satiety hormonal regulation [59].

In addition, the gut microbiota may have a significant role in satiety control. In healthy rats undergoing different food access and exercise interventions, *Lactobacillus* and *Bifidobacterium* genera were associated positively to leptin and negatively to ghrelin levels, suggesting that regulatory mechanisms of gut microbiota may influence food energy harvesting and body weight [60]. However, in disease states, gut microbiota and satiety mediators as ghrelin have different effects; in patients with polycystic ovary syndrome, ghrelin levels were negatively correlated with chronic inflammation-related gram-negative bacteria as *Bacteroides*, *Escherichia*/*Shigella* and *Blautia* species, and positively correlated with *Akkermansia* species, which are involved in maintaining intestinal integrity [61]. In children with acute lung injury, the administration of oral *Lactobacillus acidophilus* over 10 days increased ghrelin levels and decreased inflammation markers, suggesting a regulatory effect of gut microbiota on ghrelin levels [62]. Total ghrelin levels are increased in CKD patients, as ghrelin is mainly degraded by kidneys; however, this increase associates with an increase in obestatin and desacyl forms of ghrelin, which may induce effects that are opposite to those of the active orexigenic form of ghrelin, i.e., acyl ghrelin, thus decreasing appetite. On the other hand, serum leptin, the counteracting hormone of ghrelin, increases with the decrease in renal function, exacerbating the anorexigenic effect [63]. Nonetheless, there is a lack of studies on the impact of gut microbiota alterations on the levels of leptin and different forms of ghrelin, and on appetite in CKD patients.

Additionally, it has been suggested that microbiota-derived proteins may have a direct influence on satiety control. Administration of an *Escherichia coli* derived protein, caseinolytic protease, resulted in decreased food intake in mice, through neuronal central effects by activating the melanocortin-4 receptor and stimulating release of satietogenic gut hormones as glucagon-like peptide-1 (GLP-1) and peptide YY (PYY) [64]. CKD patients, including those undergoing PD, show an increase in cholecystokinin and peptide YY, leading to early satiety and decrease in nutrient intake [65, 66]. Consequently, it is possible that the observed abundance of gram-negative bacteria (Proteobacteria) in patients on PD [17] might contribute to increased appetite abnormalities.

Amino acid imbalances, characterized by low levels of branched chain amino acids, are related to an anorexic pattern in uremia; serotonin production is increased along with higher availability of tryptophan in cerebrospinal fluid, thus suppressing appetite; this phenomenon is enhanced by inflammation and metabolic acidosis [59]. Serotonin balance may be altered by the availability of tryptophan and interactions among host-bacteria (diet, drugs); gut microbiota may affect serotonin availability by increasing tryptophan degradation (tryptophanase), by synthesizing tryptophan (tryptophan synthase) or by producing de novo serotonin direct from tryptophan. A wide range of bacterial families are involved in serotonin biosynthesis, including *Bifidobacterium*, *Streptococcus*, *Escherichia*, *Enterococcus*, *Lactococcus*, *Lactobacillus* and *Clostridium* [27, 67].

On the other hand, SCFAs have an anorectic effect by stimulating intestinal GLP-1 and PYY, and decreasing ghrelin release in the context of obesity, however, it seems that the appetite suppression effect occurs only with a high and acute dose of fiber or SCFAs [57]. In CKD, inflammation and uremic toxin retention are well recognized causes of PEW by inducing anorexia, decreasing nutrient intake, and increasing resting energy expenditure as well as muscle and fat catabolism [10, 68]. It is possible that the anti-inflammatory effect of SCFAs could be more important than the appetite suppressant and energy expenditure effect in preventing PEW, however, this warrants further research.

Current studies mainly focus on the relationship between gut microbiota, appetite and weight regulation in obesity. The results of these studies may suggest possible new therapeutic targets also in patients with under-nutrition conditions, such as PEW.

## Gut microbiome manipulation and PEW in CKD

Modification of gut microbiota through nutritional and lifestyle interventions seems promising in CKD [69]. Studies in cancer cachexia, obesity, and aging settings show that modifications of gut microbiota using exercise or dietary means—including nutraceutical supplements with prebiotics, probiotics and synbiotics, or postbiotics (i.e., specific products from live bacteria such as butyrate)—may improve markers of muscle wasting; however, these studies have been carried out mostly in animal models and to date, there are few clinical trials [37].

To the best of our knowledge, no clinical trials involving CKD patients have attempted to specifically evaluate the effect of gut microbiota modification on PEW outcomes. Probiotics, prebiotics and symbiotics have been studied in CKD patients as a means to decrease inflammation and uremic toxins; however, there is limited evidence and results are controversial [70]. Nevertheless, a few studies analyzed nutritional status parameters related to the PEW syndrome. In a clinical trial to evaluate the effect of probiotics on glycemic control and oxidative stress in diabetic HD patients, the administration of a mixture of *Lactobacillus acidophilus*, *Lactobacillus casei* and *Bifidobacterium bifidum* over 12 weeks resulted in significant improvements in nutritional status as evaluated by means of subjective global assessment [71]. Intake of a mixture of 40 g of fermentable carbohydrates (inulin and potato starch) for 5 weeks, significantly increased calorie and protein intake, as well as body weight in CKD patients [72]. In HD patients, administration of resistant starch (16 g/day) increased calorie and fat intake compared to the placebo group [73]. In PD patients, the administration of a probiotic mixture (*Bifidobacterium longum*, *Lactobacillus bulgaricus*, and *Streptococcus thermophilus*) for two months increased adiposity parameters such as mid-arm circumference and triceps skinfold, as well as serum albumin [74]. Finally, Viramontes-Hörner et al*.* [75] found a significant reduction in frequency and severity of gastrointestinal symptoms in HD patients after 2 months of administration of a symbiotic product (inulin + *Lactobacillus acidophilus* and *Bifidobacterium lactis*), as well as a trend towards improvement of PEW (as assessed by subjective global assessment) in spite of a lower calorie intake. Several limitations of the previous interventional studies must be emphasized: nutritional parameters were evaluated as secondary outcomes, PEW was not specifically diagnosed, interventions were not homogeneous (either prebiotics or probiotics), and it was not clearly stated whether these interventions were able to beneficially modify gut microbiota, and subsequently improve nutritional parameters. Future research is warranted to clarify these issues.

## Conclusions

Gut microbiota disturbances may contribute to PEW in CKD. Interactions between gut dysbiosis and PEW in patients with CKD are complex and involve many interlinked factors such as inflammation, retention of uremic solutes and hormonal abnormalities. Available evidence suggests bidirectional communication between gut microbiota and key factors of PEW, such as energy balance, appetite, nutrient intake, and muscle metabolism. There are many gut-derived compounds related to dysbiosis that may influence PEW development in CKD. Altogether these findings suggest that interventions to manipulate the intestinal microbiome could be an efficient strategy to improve nutritional status and thereby clinical outcomes in CKD patients. Further studies are warranted in this as yet largely unexplored field.

## Data Availability

This is not applicable for this type of review manuscript as it does not involve any data.
